# Universal scaling of robustness of ecosystem services to species loss

**DOI:** 10.1038/s41467-021-25507-5

**Published:** 2021-08-27

**Authors:** Samuel R. P.-J. Ross, Jean-François Arnoldi, Michel Loreau, Cian D. White, Jane C. Stout, Andrew L. Jackson, Ian Donohue

**Affiliations:** 1 Department of Zoology, School of Natural Sciences, Trinity College Dublin, Dublin, Ireland; 2 Theoretical and Experimental Ecological Station, CNRS, Moulis, France; 3 Department of Botany, School of Natural Sciences, Trinity College Dublin, Dublin, Ireland

**Keywords:** Ecosystem ecology, Theoretical ecology, Ecosystem services

## Abstract

Ensuring reliable supply of services from nature is key to the sustainable development and well-being of human societies. Varied and frequently complex relationships between biodiversity and ecosystem services have, however, frustrated our capacity to quantify and predict the vulnerability of those services to species extinctions. Here, we use a qualitative Boolean modelling framework to identify universal drivers of the robustness of ecosystem service supply to species loss. These drivers comprise simple features of the networks that link species to the functions they perform that, in turn, underpin a service. Together, they define what we call network fragility. Using data from >250 real ecological networks representing services such as pollination and seed-dispersal, we demonstrate that network fragility predicts remarkably well the robustness of empirical ecosystem services. We then show how to quantify contributions of individual species to ecosystem service robustness, enabling quantification of how vulnerability scales from species to services. Our findings provide general insights into the way species, functional traits, and the links between them together determine the vulnerability of ecosystem service supply to biodiversity loss.

## Abstract (web summary)

Responses of ecosystem services to species losses are highly context-dependent. Here, the authors develop a model to identify general rules in the robustness of ecosystem service supply to species losses, and demonstrate its applicability using real-world ecosystem service networks.

## Introduction

Ecosystem services—ecological structures, functions or processes that directly or indirectly contribute to human wellbeing^1^—provide a plethora of benefits to humanity^2,3^. Through current global environmental change, humans are driving species to extinction at rates hundreds to thousands of times in excess of background^4–6^. Such extinctions compromise the capacity of ecosystems to reliably provide the goods and services upon which human societies depend^7–13^. The mechanisms through which ecosystem services collapse—that is, when they are either no longer being supplied or have declined to the point that they are no longer utilised by people (that is, are no longer in demand)—remain elusive, however, and vary with both ecological and social context^14–20^. Consequently, we have only a rudimentary understanding of the vulnerability of many, perhaps all, ecosystem services to species loss^8,12,21–23^. Certainly, different services vary in their vulnerability^24^, but we lack general rules that might explain these differences.

**Fig. 1:**
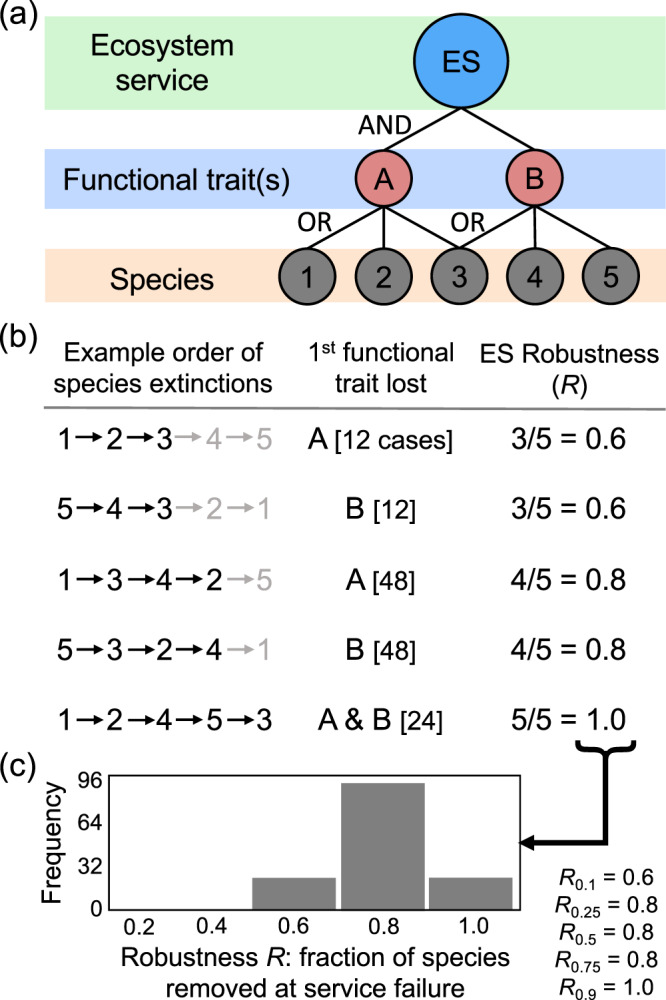
The principles of our approach to measuring the robustness of ecosystem services to species loss. **a** We use a qualitative framework (see ‘Methods’ for a detailed description) whereby species [1–5 in this example] are linked to their functional traits [A and B] and are either present or absent from a community. An ecosystem service [ES] is supplied only when all underpinning functional traits are present in the community (the Boolean function AND; see ‘Methods’ and Supplementary Methods for justification). **b** As species are removed sequentially from the system, a functional trait is present until all species connected to that trait are lost. In this way, species losses are tolerated (the Boolean function OR) until a unique functional trait has been lost. ‘Example order of species extinctions’ presents illustrative examples of the sequential removal of the species in **a**, with sequences in grey indicating extinctions after service failure. ‘1^st^ functional trait lost’ is the identity of the unique functional trait [A and/or B] first lost from the system—which, under our framework, necessarily causes service loss—with [*n* cases] indicating the number of extinction sequences for which this result is obtained [reflected also in the histogram (**c**)]. We measure ‘ES Robustness (*R*)’ as the fraction of species removals required to bring about loss of a service for each extinction sequence. **c** This results in a distribution of robustness *R* values, representing the fraction of species loss tolerated across all extinction scenarios. This distribution of *R* values can be described via its percentiles [denoted with subscript *c*] with, for example, the median robustness [*R*_0.5_] here equating to 0.8. That is, in 50% of extinction scenarios in this hypothetical example, 4/5 or fewer species extinctions are required to cause service failure.

**Fig. 2:**
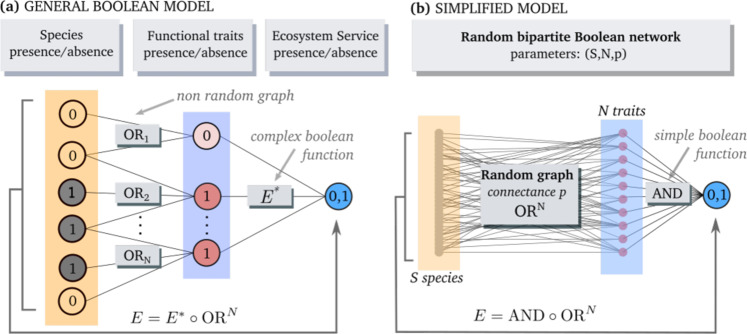
A Boolean model for ecosystem services. **a** We view an ecosystem service as a Boolean function *E* over the presence/absence configurations of *S* species (leftmost column). We distinguish species from their *N* functional traits (middle column). The configuration of any trait *n* is defined by the logical OR function over the *S*_*n*_ species that share that trait. This amounts to writing *E* as the composition $E={E}^{\ast }\circ {{{{{\rm{OR}}}}}}^{N}$, which should aim to remove as much functional redundancy as possible from the auxiliary function ${E}^{\ast }$ over trait configurations. **b** We simplify our model further by (i) considering random species-to-trait associations with connectance *p* and (ii) choosing the logical AND function for ${E}^{\ast }$ [that is, the least redundant function, which we show (Supplementary Methods) is representative of a random choice across the space of all Boolean functions]. In this model (see ‘Methods’ for full description), we can deduce analytical expressions for the percentiles of the distribution (considering all possible extinction sequences) of robustness of ecosystem service supply *R*: the fraction of species loss leading to loss of service supply.

Here, we develop and test a simple model to measure and predict the robustness of ecosystem service supply to species loss. We take a purely qualitative perspective, where species are either present or absent and an ecosystem service is provided or not (Figs. 1a and 2a). We focus on the implications of species removal and aim to understand how functional redundancy and species richness combine to buffer ecological processes against biodiversity loss^25–27^. We limit our scope to the supply of ecosystem services and do not address here the extent or dynamics of human demand for those services. On the other hand, our approach could be applied to any emergent, high-level ecosystem process, not necessarily considered (or recognised) as a service.

We use Boolean functions to model a robust–vulnerable continuum^28^ of ecosystem services (Fig. 2). At one extreme is the logical AND function, where every species is essential to the supply of an ecosystem service. In this case, even the loss of a single species will result in service failure, owing to a complete lack of functional redundancy^29^. At the other extreme, all species are fully substitutable and only a single species is then needed to supply a service. In this case, the ecosystem service equates to the logical OR function, where full redundancy among species promotes robust service supply even in the face of multiple species extinctions^25,29,30^.

For a given ecosystem service, rather than consider a particular model of extinction scenarios, we instead consider all possible extinction sequences simultaneously (Fig. 1b). We study the entire distribution of robustness, defined as the fraction of extinctions—along all extinction scenarios—sufficient to bring about the loss of supply of the ecosystem service (Fig. 1c). Services that can, along most extinction sequences, tolerate a high number of extinctions before they collapse are the most robust. We assume that the supply of a service requires species that have particular traits—phenotypic attributes that direct niche exploitation—whose presence drive all of the various ecological processes that are necessary to underpin provision of the service^17,31^. Though many traits, such as body mass, occur along a continuous spectrum^31–33^, for simplicity we focus here only on whether or not a given species possesses a particular trait^34^. We consider traits purely in a functional sense^29^, understood here as the capacity of a species to perform a particular functional role required for the ecosystem service to be provided^34^. As such, traits are functional features that can be shared among species, as can be visualised by drawing, for a given service, a bipartite network linking species to traits^19^ (Figs. 1a and 2, but note that we could, in principle, refine the notion of traits by decomposing species into populations and differentiating traits within species).

Under these simplifying assumptions^35^, we reveal the universal drivers of the robustness distribution of any ecosystem service to species loss (see ‘Methods’). We show that any percentile of the robustness distribution is driven by a synthetic parameter that we call network fragility. Network fragility combines simple features of the species-to-traits bipartite network—the numbers of species, functional traits and links between them—that together determine the fraction of species loss that can be tolerated before ecosystem service failure. We then go on to show how our results, based on properties of random bipartite networks, can be applied to non-random ecological networks representing real-world ecosystem services, such as plant pollination and seed dispersal.

## Results

In our framework, an ecosystem service *E* consists of *N* required functional traits shared between *S* species, with *p* characterising the connectance of the species-to-traits network (Fig. 2). We note *R*(*E*), the distribution of robustness, that is, the distribution of the fraction of species loss along all extinction sequences that lead to ecosystem service failure (Fig. 1c). For random species-to-traits networks, we find that the *c*th percentile *R*_*c*_(*E*) of *R*—the value *R*_*c*_ such that a fraction *c* of extinction sequences leads to service failure when losing less than *S* × *R*_*c*_ species (Fig. 1c)—takes the analytic form (if *q* = 1 − *p*; see Supplementary Methods)

1 $${R}_{c}(E)=1-{f}_{c};\,{{{{{\rm{where}}}}}}\,{f}_{c}=\frac{\log (1-(1-{q}^{S}){e}^{-\frac{c}{N}})}{S\,\log \,q}$$

**Fig. 3:**
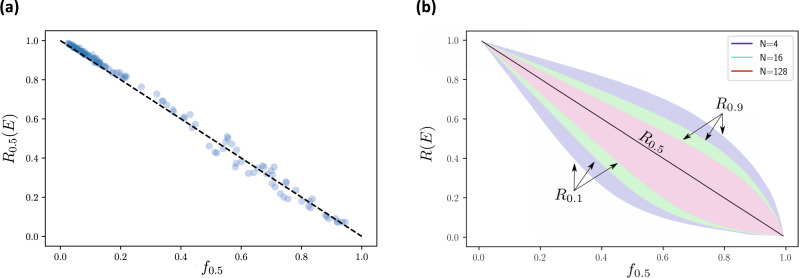
Universal behaviour of the robustness of ecosystem service supply as a function of network fragility. **a** For 125 random ecosystem services of the form shown in Fig. 2, we uniformly sampled 5 values for species richness *S* between 10 and 200, 20 for functional traits *N* between 10 and 100 and 10 for connectance *p* between 0 and 0.5 and for each considered 600 extinction scenarios leading to a loss of service, drawn uniformly over the set of all possible extinction sequences. We see that the median robustness of ecosystem service supply (that is, *R*_0.5_(*E*)) in our simplified Boolean model behaves as a simple decreasing linear function of median network fragility (*f*_0.5_). **b** For three values of number of traits (that is, *N* = 4, 16 and 128) and random values of number of species *S* and connectance *p*, we used the analytical formula in Eq. (1) to draw the contours of the bulk of the robustness distribution (that is, 10th–90th percentiles) as a function of *f*_0.5_, the value of fragility associated with median robustness. We see that, at fixed *N*, the percentiles can be expressed as functions of *f*_0.5_, with the largest spread at intermediate values of network fragility. At fixed fragility, increasing the number of traits *N* reduces the width of the distribution.

The statistics of *R*(*E*) are, therefore, driven entirely by a family of parameter values (*f*_*c*_), comprising measures of what we call network fragility. In particular, the median value for robustness of ecosystem service supply corresponds to *f*_0.5_ (Fig. 3a). The width of the distribution of *R*(*E*) is maximal at intermediate values of *f*_0.5_ and decreases as the number of traits *N* increases (Fig. 3b). This means that the robustness of ecosystem service supply to species loss is at its most predictable at both low and high values of network fragility and when the service is underpinned by many functional traits.

Next, we examine whether the one-to-one relationship between network fragility and the statistics of robustness of ecosystem service supply is universal, that is, valid beyond random species-to-traits association networks. To do this, we considered 251 real empirical bipartite networks from the Web of Life ecological networks database, representing a variety of ecosystem services, including, for example, pollination and seed dispersal (see ‘Methods’). We measured network fragility for each network directly from the numbers of species, traits and links it comprises. To measure the robustness of ecosystem service supply, we simulated random extinction sequences leading to the loss of at least one functional trait [for example, the loss of pollination of at least one plant species following pollinator species extinctions (see ‘Methods’ and Supplementary Methods)] and replicated this process 1000 times to give a numerical estimate of the distribution of *R* for each service represented by each empirical network. In addition, we computed the variance in the number of species per trait, ${\mathbb{V}}$(*S*_*n*_), which we compared to a null expectation, ${\mathbb{V}}$_0_(*S*_*n*_), if the species-to-traits network were random [for a random network with connectance *p*, ${\mathbb{V}}$_0_(*S*_*n*_) = *Spq*]. We then defined dispersion *d* as

2 $$d=\frac{{\mathbb{V}}({S}_{n})}{{{\mathbb{V}}}_{0}({S}_{n})}$$

so that log_10_(*d*) gives a measure of deviation from randomness, vanishing if the observed variance is that of a random network, is negative if the variance is smaller and positive if it is larger.

**Fig. 4:**
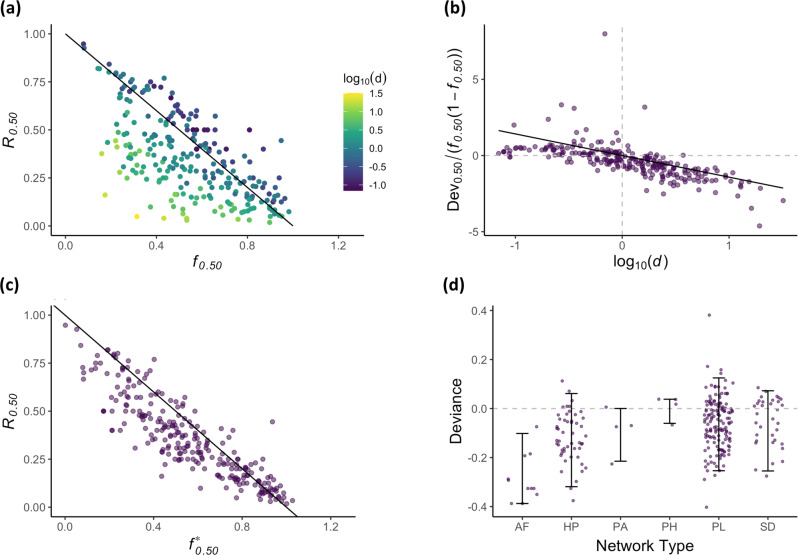
Robustness of ecosystem service supply and network fragility in empirical networks. **a** The relationship between simulated robustness of ecosystem service supply (*R*_*c*_ = *R*_0.5_) and analytically estimated network fragility (*f*_*c*_ = *f*_0.5_) for 251 empirical networks from the Web of Life follows the linear approximation *R*_*c*_ = 1 − *f*_*c*_. Colour indicates network dispersion [log_10_(*d*)]. **b** The residuals off the linear prediction *R*_*c*_ = 1 − *f*_*c*_ correlate with log_10_(*d*), which we model as (*R*_*c*_ − 1 + *f*_*c*_)/(*f*_*c*_(1 − *f*_*c*_)) = ${\lambda }_{c}$log_10_(*d*), where, for the case of *R*_0.5_, ${\lambda }_{0.5}$ = −1.42. **c** Correcting network fragility for dispersion according to ${f}_{c}^{\ast }={f}_{c}+{\lambda }_{c}$log_10_(*d*) yields an even closer relationship with robustness of ecosystem service supply. **d** Residuals from *R*_0.5_ − ${f}_{0.5}^{\ast }$ by network type show that our model captures well the universal properties, where AF = anemone–fish (*n* = 10), HP = host–parasite (*n* = 51), PA = plant–ant (*n* = 4), PH = plant–herbivore (*n* = 4), PL = pollination (*n* = 148), and SD = seed dispersal (*n* = 34). Error bars show the 5th and 95th quantiles of the data.

We found that network fragility explains well the robustness of ecosystem services in the empirical networks we examined (Spearman $\rho$ = −0.64 for *c* = 0.5; Fig. 4a). However, overdispersion [that is, log_10_(*d*) > 0] led systematically to lower robustness of ecosystem service supply than predicted, whereas underdispersion [log_10_(*d*) < 0] had the opposite effect. Correcting network fragility (${f}_{c}^{\ast }$) to then account for the deviation from randomness of the species-to-trait network (Fig. 4b), where

3 $${f}_{c}^{\ast }={f}_{c}+{\lambda }_{c}\;{f}_{c}(1-{f}_{c}){\log }_{10}(d),{{{{{\rm{for}}}}}}\,{\lambda }_{c} > 0$$

improved the relationship with robustness of ecosystem service supply considerably (Spearman $\rho$ = –0.89 for *c* = 0.5; Fig. 4c) and captures well its universal properties (Fig. 4c, d and Supplementary Fig. 1). Moreover, we confirmed that uncertainty in ecosystem service robustness in the empirical networks was greatest at intermediate values of network fragility (Supplementary Fig. 2), as predicted by our analytical model (Fig. 3b). Variance among the different types of empirical networks in our analysis (Fig. 4d) suggests that there may be further nuances in the structure of those networks that contribute to their robustness to species loss beyond the universal characteristics we describe. However, these unknown drivers play a minor role in comparison to network fragility.

Whereas the distribution of robustness reflects the complex topology of species presence–absence configurations over which a given ecosystem service is supplied, network fragility can be evaluated directly from the most basic features of species-to-traits associations—the numbers of species, functional traits that underpin the service and links between them. This local nature of network fragility therefore allows us to study its behaviour under any specific species extinction scenario. This crucial property allows us to quantify the contribution of any given species or combination of species to the robustness of ecosystem service supply. We can, for example, ask how much network fragility ${f}_{c}^{\ast }$ would increase if species *i* were lost? The loss of a species with *L*_*i*_ traits will obviously reduce richness $S\to S-1$, but it will also affect connectance as

4 $$p\to p-p\left(\frac{{L}_{i}}{L}-\frac{1}{S}\right)$$

Naturally, the species with the most links will reduce connectance, and therefore increase network fragility, the most. Species loss may, however, also affect dispersion *d* via its effect on the variance in the number of species per trait ${\mathbb{V}}$(*S*_*n*_). If we define $\overline{{S}_{{n}_{i}}}$, the mean number of species that have the same traits as species *i*, and $\overline{{S}_{n}}$ = *L*/*N* the mean number of species per trait, we get that (approximately)

5 $${\mathbb{V}}({S}_{n})\to {\mathbb{V}}({S}_{n})+2\overline{{S}_{n}}\,(\overline{{S}_{n}}\,-\overline{{S}_{{n}_{i}}})\frac{{L}_{i}}{L}$$

Thus, dispersion (and, therefore, network fragility) increases the most if $\overline{{S}_{{n}_{i}}}$ is small compared to $\overline{{S}_{n}}$, that is, if the traits of species *i* are uncommon. It is, therefore, those species that are generalist providers of multiple uncommon traits that contribute the most to the robustness of ecosystem service supply. This is an intuitive statement, but our theory makes it a quantitative measure, integrating precisely the relevant features of the service to which a given species contributes.

## Discussion

Our model reveals how species richness and trait generality combine to determine the robustness of ecosystem service supply to the loss of species. Moreover, by also enabling quantification of the contribution of individual species to the robustness of ecosystem service supply, the model comprises a general tool for predicting and managing the vulnerability of ecosystem service provision (or, indeed, any ecological process) in the face of rapid and ongoing global environmental change.

Our theory predicts that extinction scenarios targeting species that possess multiple uncommon traits will likely lead to more rapid collapse of ecosystem service supply. That is, a species with unique links in a bipartite species-to-traits network is more likely to contribute substantially to robustness^36^. As a network transitions via sequential extinctions from a starting point of high robustness, it will, however, pass through a window at intermediate network fragility where uncertainty in robustness is maximal. Thus, increasing network fragility via loss of species (or loss of their functional roles) not only reduces the robustness of service supply but also increases the uncertainty surrounding predictions of ecosystem service loss in the face of uncertain species extinction scenarios.

The theory we present comprises a highly simplified representation of nature—our model assumes that ecosystem services are either provided or not and that the loss of a single unique functional trait causes ecosystem service provision to cease. The latter assumption may seem to be overly conservative, yet we show (Supplementary Methods) that it captures well the robustness of randomly generated services. In addition, we did not consider either species abundances—which did not stabilise crop pollination services in a previous study^30^—or intraspecific trait variation, which often supports ecosystem service supply^37^. Such detailed knowledge could, however, easily be incorporated in our model by replacing species with populations or even individuals. The resulting network representation of the service would then become highly complex^19^. Importantly, however, our notion of network fragility would still apply and, because of its simplicity, would remain tractable. All in all, our abstractions of natural systems enabled us to gain general insights into biodiversity–ecosystem services relationships, taking a significant step forward in our understanding of the stability of ecosystem service provision in the face of widespread biodiversity loss.

Though biodiversity is foundational to the sustained supply of ecosystem services, ecological factors alone do not determine their overall vulnerability^38^. Ecosystem services are linked intrinsically to human valuation^1,2,35^, and shifts in their value can play an overarching and even dominant role^39,40^. The overall vulnerability of ecosystem services may, therefore, depend on the stability of human values to an even greater extent than on biodiversity. Models linking the ecological and social aspects of ecosystem services, and the interactions between them, are, therefore, fundamental to understanding how ecosystem services will respond to future changes^7,19,20,40,41^. This delineates a challenge for future research: how can we best integrate the robustness of ecosystem service supply with the various drivers of human valuation and demand? Our modelling framework serves as a stepping stone in that direction.

By enabling identification of the key species that contribute to the robustness of ecosystem service supply, our theory provides a focus for management efforts that require consideration of the conservation value of individual species^8,10,32,34,36,42–44^. Focussing on the contribution of individual species to robustness and the vulnerability of those species to extinction through their life histories, interactions and functional traits^24,31,45,46^ will enable empirical quantification of how vulnerability scales from species through to ecosystem services^11,12,15,18,43^. Ultimately, such a shift will provide a far richer and mechanistic understanding of the sustainability of nature’s contributions to people under global environmental change.

## Methods

### A Boolean model for the robustness of ecosystem service supply

We view the supply of an ecosystem service as a Boolean function *E* over the presence/absence configurations of *S* species

6 $$E:{\{0,1\}}^{S}\to \{0,1\}$$

We consider here only the presence and absence of species, though one could replace species with individuals to explore population-level contexts. Ecosystem service provision is also considered as binary—a service is either provided or it is not. Though these assumptions clearly simplify reality^35^, they nonetheless provide the foundation from which general insights applicable to real ecosystems can be developed, as we will show.

We are interested in the robustness *R*(*E*) of supply of a given ecosystem service *E*. That is, the distribution—considering all possible extinction sequences—of the fraction of species extinctions that leads to loss of ecosystem service supply. Robustness is maximal and reduced to a single number if the service is lost only when all species are extinct [*R*(*E*) ≡ 1], and minimal and reduced to a single number if any species extinction is sufficient to cause service failure [*R*(*E*) ≡ 1/*S*]. More formally, if we consider all extinction scenarios, *R* is a distribution over the range $[\frac{1}{S},1]$. This distribution encodes the complex topology of species presence–absence configurations over which an ecosystem service is supplied. Considering the proportion of extinctions (rather than the absolute number) that result in service failure allows us to compare across systems of different species richness. It also leads to a notion of ecosystem service robustness that relates directly to the expected time to service loss, given a mean species extinction rate (Supplementary Methods).

Next, we distinguish species from their *N* potential functional traits that relate to the ecosystem service of interest. We model functional redundancy by determining the configuration of trait *n* with the Boolean function OR over the configuration space of the *S*_*n*_ species that share this trait (Fig. 2a). Thus, a trait is present until all species that have this trait are extinct. If we denote OR_*n*_ as the extension of the OR function over the *S*_*n*_ species that share a given trait to the whole species configuration space (that is, a partial OR function), we have a mapping from *S* species configurations to *N* traits configurations:

7 $${{{{{{\rm{OR}}}}}}}^{N}={{{{{{\rm{OR}}}}}}}_{1}\times \ldots \times {{{{{{\rm{OR}}}}}}}_{N}:{\{0,1\}}^{S}\to {\{0,1\}}^{N}$$

The state of the ecosystem service *E* is then determined by an auxiliary Boolean function ${E}^{\ast }:{\{0,1\}}^{N}\to \{0,1\}$ over trait configurations. In other words, we factorise *E* as the composition

8 $$E={E}^{\ast }\circ {{{{{{\rm{OR}}}}}}}^{N}:{\{0,1\}}^{S}\to {\{0,1\}}^{N}\to \{0,1\}$$

This factorisation is always possible, since by choosing *N* = *S* we could always make OR^*N*^ into a trivial mapping where each species has a unique trait and take ${E}^{\ast }\equiv E$. To be useful, however, this factorisation should aim at having the lowest number of distinct functional traits relative to the service, thus removing as much functional redundancy as possible from the auxiliary function ${E}^{\ast}$.

To derive generic drivers of the robustness of ecosystem service supply, we need to define a universe of ecosystem services rich enough to be representative of real ecosystem services, yet simple enough to permit an analytical treatment of their robustness. Based on our factorisation [Eq. (8)], there are two parts to the problem, and we make simplifying assumptions for both. We start by defining a universe of species-to-traits mapping. A simplifying choice is to assume the species-to-traits network to be a random graph with connectance *p*. We then need a model for the auxiliary function ${E}^{\ast }$. A natural choice, given our treatment of the species-to-trait association, would be to pick ${E}^{\ast }$ at random and uniformly over the set of all ${2}^{{2}^{N}}$ possible Boolean functions over trait configurations. However, we can make a much simpler choice at a low cost in behavioural complexity. In terms of robustness of ecosystem service supply, we find (by drawing the auxiliary function ${E}^{\ast }$ at random uniformly over the set of all Boolean functions of {0,1}^*N*^; Supplementary Methods) that assuming the AND function (that is, the least redundant function) for ${E}^{\ast }$ is representative of the outcome of a random choice. Thus, we now have, for any choice of *S*, *N* and *p*, a random set (the random component is in the species-to-trait graph, Fig. 2b) of ecosystem services of the form

9 $$E={{{{{\rm{AND}}}}}}\circ {{{{{{\rm{OR}}}}}}}^{N}:{\{0,1\}}^{S}\to {\{0,1\}}^{N}\to \{0,1\}$$

This then allows us to derive an analytic form for the robustness of ecosystem service supply (Supplementary Methods).

### Analysis of empirical networks

For our analysis of empirical networks, we downloaded all 258 available bipartite webs from the Web of Life ecological networks data set (http://www.web-of-life.es), which comprised anemone–fish (*n* = 17), host–parasite (*n* = 51), plant–ant (*n* = 4), plant–pollinator (*n* = 148) and seed–disperser (*n* = 34) networks. We removed three networks that comprised only a single trait (that is, where all species connected to the same single interaction partner) and four that showed no variation in the number of species per trait (of which the latter were all anemone–fish webs), leaving 251 networks for analysis. We converted the count-based association matrix to binary so that connections between species and traits were either present or absent. In these networks, the ‘traits‘ are also species. For example, we view pollinators as the species that provide for the presence of a plant^13^, which is a web-level trait. These web-level traits then underpin the provision of the overall pollination service, which we consider here to be the pollination of all plants in the network. That is, in order for there to be an arbitrarily defined pollination service for this ecosystem^47^, we demand that all plants (traits) are present so that, if one plant is lost, then the pollination service is lost. In contrast, the pollinators themselves are compensable for a given plant (trait) and so species loss is tolerated if there remains another species to pollinate that plant^48^. Similarly, we have chosen to view the networks as trait–species such that seed–disperser refers to plant species as the trait and disperser as the species that provides for that trait. That these networks really are or are not ecosystem services, or whether some species would go extinct together due to interactions^15^, is not relevant for our purpose. We simply use these empirical networks as a rich set of non-random example networks on which to showcase the relevance of our theory beyond random networks.

For each of the binary bipartite webs, we calculated network fragility ${f}_{c}$ and dispersion-corrected network fragility ${f}_{c}^{\ast }$ using, respectively, Eqs. (1) and (3). Connectance *p* was estimated as *L*(*S* × *N*), where *L* is the number of links. The distribution of robustness of ecosystem service supply *R* was then determined by simulation, whereby species were selected at random and removed sequentially until a trait (and hence the service; Supplementary Methods), was lost and *R* was then quantified as the proportion of species lost until service collapse (Fig. 1). By simulating sequential extinctions and measuring robustness as a distribution in this way, our model encompasses all possible combinations of extinction orders, which is critical given the non-random nature of species extinctions in real ecosystems^49–51^.

### Reporting summary

Further information on research design is available in the Nature Research Reporting Summary linked to this article.

## Supplementary information

Supplementary Information

Reporting Summary

## Data Availability

The species–traits network data generated in this study have been deposited in Zenodo under accession code 10.5281/zenodo.4749405 (ref. ^52^). The empirical network data are publicly available from the Web of Life ecological networks database (http://www.web-of-life.es).

## Code availability

Simulations and data analyses were performed in R. All code necessary to reproduce the results are publicly accessible from Zenodo: 10.5281/zenodo.4749405 (ref. ^52^).
