# Supplementary material for: Universal scaling of robustness of ecosystem services to species loss
Source: Nat Commun. 2021 Aug 27;12:5167. doi: 10.1038/s41467-021-25507-5 (PMC8397752; doi:10.1038/s41467-021-25507-5)
Supplement: Supplementary file 1 — Supplementary Information [file 41467_2021_25507_MOESM1_ESM.pdf]

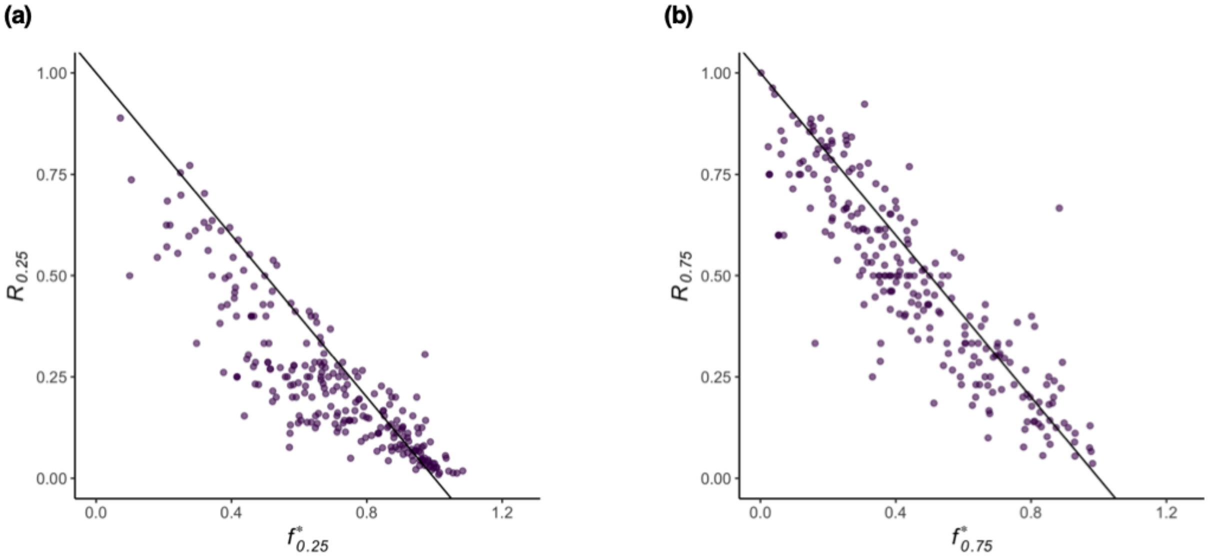

Supplementary Fig. 1 | Relationships between simulated robustness of ecosystem service supply quartiles R_c and analytically-estimated network fragility f_c^* where c = (a) 0.25 (for which $\lambda_{0.25}$ = -1.65) and (b) 0.75 (for which $\lambda_{0.75}$ = -1.35), from the analysis of 251 empirical networks as described in Fig. 4.

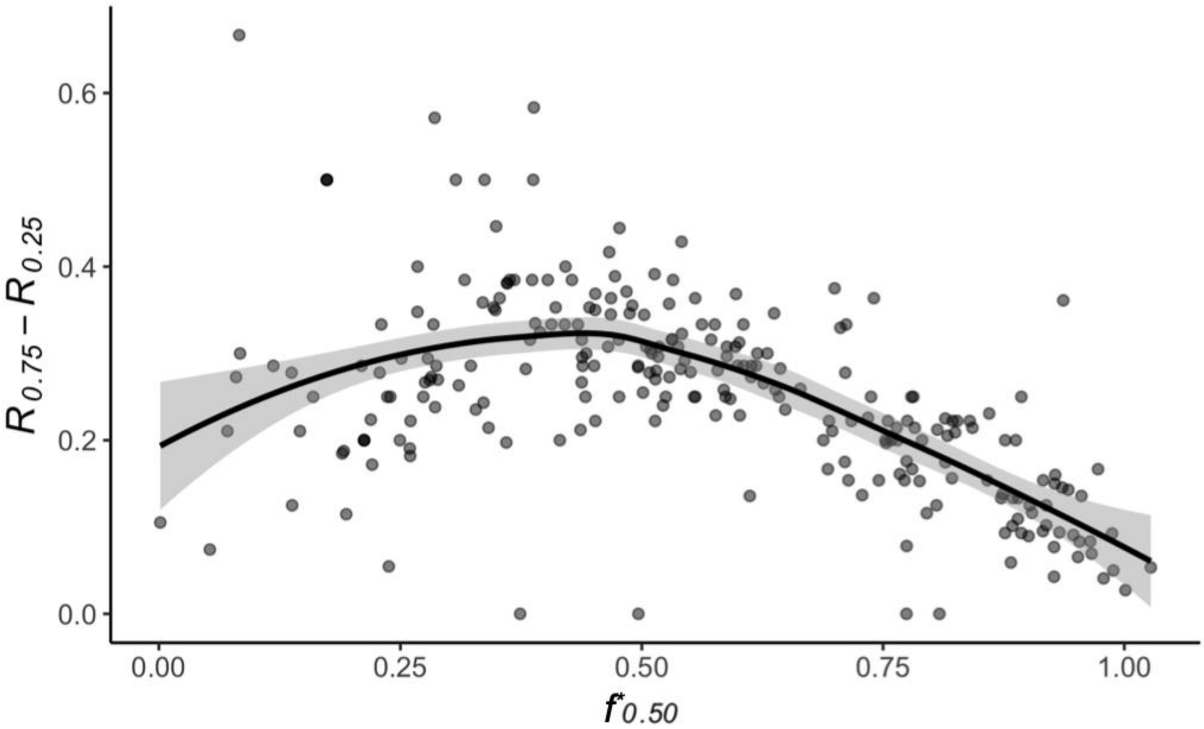

Supplementary Fig. 2 | Uncertainty in robustness of ecosystem service supply calculated from 251 empirical networks is greatest at intermediate values of network fragility (*$f^*_{0.5}$*). We quantified uncertainty as the interquartile range $R_{0.75} – R_{0.25}$. A loess smoother (span = 0.8) with 95% confidence intervals is shown to illustrate the concave relationship.

Supplementary Methods for

Universal scaling of robustness of ecosystem services to species loss

Samuel R. P-J. Ross, Jean-François Arnoldi, Michel Loreau, Cian D. White, Jane C. Stout, Andrew L. Jackson, Ian Donohue

Contents

S1 Robustness in the simplified model **1**

S2 Time to service failure **3**

S3 Random Boolean Model **3**
S3.1 Redundancy of random Boolean functions 3
S3.2 Simulation of the random model 4

S1 Robustness in the simplified model

In the main text (see Methods), we describe an analytically tractable model of ecosystem processes (e.g. services), seen as Boolean functions of the presence/absence of S species (but one could think of populations or even individuals). We distinguish species from their N functional traits, and consider a special set of Boolean functions of the form

$$E = \text{AND} \circ \text{OR}^N : \{0,1\}^S \to \{0,1\}^N \to \{0,1\}$$

where $\text{OR}^N : \{0,1\}^S \to \{0,1\}^N$ is a mapping from species to traits configurations. It is the Cartesian product of N (partial) OR functions associated with traits (thus, a trait is present if at least one species that has this trait is present). We model the universe of OR^N as random bipartite networks with connectance p. Deriving an expression for the distribution of fraction of species extinctions that lead to a loss of service is the goal of this subsection. We start from the probability of having lost at least one trait when n species are lost. The latter reads

$$P(n_e \leq n) = 1 - (1 - q^{S-n})^N; \; q = 1 - p \tag{S1}$$

where n_e would be the number of extinctions that led to the loss of the first trait. If we write $p(n_e)$, the probability of the loss occurring after exactly n_e extinctions

$$P(n_e \leq n) = \sum_{n_e=1}^{n} p(n_e) \tag{S2}$$

We can understand Equation (S1) from the following argument. Consider one functional trait and write its distribution over species as a vector

$$[\underbrace{0,0,0,}_{S-n}\underbrace{1,...,0,1}_{n}]$$

Extinctions proceed from the right, removing entries until only the $S-n$ last entries remain. The trait is still represented at this stage if not all remaining entries are zero. The probability of all these entries being zero is q^{S-n} and, thus, the probability of the trait still being present is $1-q^{S-n}$. That all traits are present then reads $(1-q^{S-n})^N$, so that the complementary probability is the probability that at least one trait is lost after n extinctions, thus that $n_e \leq n$.

We have not yet conditioned on the fact that the process ought to be running when all species are present. The probability of a trait being present before extinctions take place is $1-q^S$. The probability of all traits being present is therefore $(1-q^S)^N$, which gives us that $p(0) = 1-(1-q^S)^N$. From Bayes' theorem it holds that

$$P(n_e \leq n) = p(n_e \leq n | n_e = 0)p(0) + P(n_e \leq n | n_e > 0)(1-p(0))$$

By definition, $P(n_e \leq n | n_e = 0) = 1$. We deduce that the conditional probability

$$P(n_e \leq n | n_e > 0) = \frac{P(n_e \leq n) - p(0)}{1-p(0)}$$

This is the cumulative probability of number of extinctions leading to the loss of at least one trait. From this expression it is straightforward to deduce any percentile of the actual probability distribution $P(n_e)$. For instance, its median $n_{0.5}$ is such that, by definition:

$$P(n_e \leq n_{0.5} | n_e > 0) = 0.5$$

More generally, the cth percentile of $P(n_e)$ is the number n_c such that

$$P(n_e \leq n_c | n_e > 0) = c$$

which we can invert to get that

$$n_c = S - \frac{\log\left(1-(1-q^S)\bar{c}^{1/N}\right)}{\log q} \quad \bar{c} = 1-c$$

We define robustness $R(E)$, along a specific extinction scenario, as the fraction n_e/S. We thus have that the cth percentile of the distribution of robustness reads

$$R_c(E) = 1 - f_c;\ f_c = \frac{\log\left(1-(1-q^S)\bar{c}^{1/N}\right)}{S\log q}$$

where we defined a family of *network fragility* measures $(f_c)_{0<c<1}$.

S2 Time to service failure

If we assume a constant rate λ of species extinction, we can deduce from the derivations in Appendix S1 the median time to functional extinction, as follows. Let p_t be the probability that a given species, present at time 0, went extinct by time t. This probability is simply

$$p_t = 1 - e^{-\lambda t}$$

On the other hand, consider the median time $t_{0.5}$ needed for $SR_{0.5}$ extinctions to occur, which, is the median number of extinctions needed to lose the function. We have that

$$Sp_{t_{0.5}} \approx SR_{0.5} \Leftrightarrow p_{t_{0.5}} = R \Leftrightarrow \lambda t_{0.5} = -\log(1 - R_{0.5}) = -\log(f_{0.5})$$

Note that this formula cannot be true at very high values of R (or low values of network fragility), where we know that an explicit logarithmic dependence in S should manifest (indeed $t_{0.5} \sim \log S$ if $R = 1$), a behaviour that takes over the simple heuristic formula (which diverges at $R = 1$).

S3 Random Boolean Model

S3.1 Redundancy of random Boolean functions

A Boolean function E^* has output 0 or 1 depending on the configuration of N bits:

$$E^* : x \in \{0,1\}^N \to \{0,1\}$$

There are 2^N bit configurations, and to each a function associates 0 or 1. Consider the case $N = 3$. The configuration space is

$$\{0,1\}^3 = \{(000), (100), (010), (110), (001), (101), (011), (111)\}$$

A function assigns a 0 or a 1 to these configurations, for instance:

$$E_0^* = [00000000]$$

assigns 0 to all configurations. It is the 0th function. In fact, to each function we associate a number which is its output list in base 2. For instance:

$$E_{69}^* = [10100010] = 1 + 0 + 2^2 + 0 + 0 + 0 + 2^6 + 0 = 69$$

is the 69th function. The last function is

$$E_{255}^* = [11111111] = 1 + 2 + 2^2 + 2^3 + 2^4 + 2^5 + 2^6 + 2^7 = 255$$

so that there are $1 + 255 = 2^8$ functions. More generally, for any N there are 2^{2^N} functions. We can uniformly sample the space of functions by assigning at random – and with equal probability – the output 0 or 1 to each configuration.

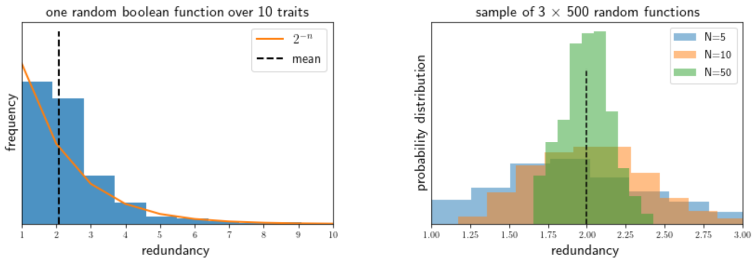

Supplementary Fig. 3. Left: redundancy of a random Boolean function over $\{0,1\}^N$, with $N = 10$. Starting from the configuration $(1, 1, ..., 1)$ the number of bitflips needed to change the function's output is exponentially distributed, with mean 2. Right: for $N = 5, 10, 50$, we sample 500 functions and plot their mean redundancy. We see that, as N grows, the distribution becomes increasingly peaked around 2. Thus, for large N, a typical function requires approximately two bitflips to change its output.

This procedure is equivalent to sampling numbers between, say, 0 and 10,000 by picking each of 4 digits from a uniform distribution over 0,1,2,...,9.

We will consider functions that have output 1 when all bits are 1, and 0 when all bits are 0.

$$E^*(1, 1, ..., 1) = 1;\ E^*(0, 0, ..., 0) = 0$$

Starting from the configuration where all bits are 1, we are interested in the redundancy of functions. That is, the number of single bit flips required (a bit is flipped at most once) to change the function output from 1 to 0. The least redundant function is the logical AND, since one bit flip is enough (redundancy= 1), the most redundant function is the logical OR, since all bits must be flipped (redundancy $= N$). From our sampling procedure we deduce that, for a random function, the probability that its output changes after $n$flips is 2^{-n}, and the probability that it takes all bits to be flipped is $2 \times 2^{-(2^N-1)}$ (and not $2^{-(2^N-1)}$ since the output must be 0 if all bits are zero; we can check that, with this constraint, the probability sums to one over the $2^N - 1$ bit flips). We deduce that, for a typical function, redundancy is exponentially distributed with an average of two. Over a uniform sampling of functions their mean redundancy will distribute around two, with the distribution becoming increasingly peaked as N grows (Supplementary Fig. 3).

S3.2 Simulation of the random model

From S3.1, we expect that choosing E^* =AND (the least redundant function) over trait configurations is representative of a random choice, taken uniformly over the space of Boolean functions. Here, we demonstrate this using simulations. We generate random S-species to N-traits networks and informally

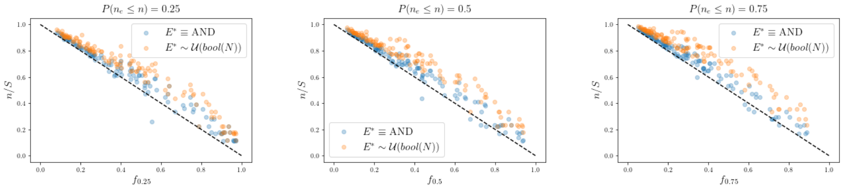

Supplementary Fig. 4. The logical AND for the auxiliary function E^* on trait configurations vs a uniform random choice over the whole space of Boolean functions bool(N). From left to right: we compare the first quantile, the median, and the third quantile of the distribution n_e/S as a function of the corresponding fragility measure ($f_{0.25}$, $f_{0.5}$ and $f_{0.75}$, respectively). As expected, the random choice shifts the distribution to slightly larger values of robustness but without qualitatively affecting its relationship with network fragility.

sample a Boolean function E^* in bool(N), the space of Boolean functions over $\{0,1\}^N$. We compare the results for robustness to the case where E^* =AND. As expected, the random choice systematically gives a larger robustness yet without qualitatively affecting the universal relationship between network fragility and robustness (Supplementary Fig. 4).
