# Supplementary material for: Universal scaling of robustness of ecosystem services to species loss
Source: Nat Commun. 2021 Aug 27;12:5167. doi: 10.1038/s41467-021-25507-5 (PMC8397752; doi:10.1038/s41467-021-25507-5)
Supplement: Supplementary file 2 — Reporting Summary [file 41467_2021_25507_MOESM2_ESM.pdf]

nature research

Corresponding author(s): Ian Donohue

Last updated by author(s): Jul 15, 2021

Reporting Summary

Nature Research wishes to improve the reproducibility of the work that we publish. This form provides structure for consistency and transparency in reporting. For further information on Nature Research policies, see our Editorial Policies and the Editorial Policy Checklist.

Statistics

For all statistical analyses, confirm that the following items are present in the figure legend, table legend, main text, or Methods section.

n/a	Confirmed	
☐	☒	The exact sample size (*n*) for each experimental group/condition, given as a discrete number and unit of measurement
☒	☐	A statement on whether measurements were taken from distinct samples or whether the same sample was measured repeatedly
☒	☐	The statistical test(s) used AND whether they are one- or two-sided<br>*Only common tests should be described solely by name; describe more complex techniques in the Methods section.*
☐	☒	A description of all covariates tested
☐	☒	A description of any assumptions or corrections, such as tests of normality and adjustment for multiple comparisons
☐	☒	A full description of the statistical parameters including central tendency (e.g. means) or other basic estimates (e.g. regression coefficient) AND variation (e.g. standard deviation) or associated estimates of uncertainty (e.g. confidence intervals)
☒	☐	For null hypothesis testing, the test statistic (e.g. *F*, *t*, *r*) with confidence intervals, effect sizes, degrees of freedom and *P* value noted<br>*Give P values as exact values whenever suitable.*
☒	☐	For Bayesian analysis, information on the choice of priors and Markov chain Monte Carlo settings
☒	☐	For hierarchical and complex designs, identification of the appropriate level for tests and full reporting of outcomes
☒	☐	Estimates of effect sizes (e.g. Cohen's *d*, Pearson's *r*), indicating how they were calculated

Our web collection on statistics for biologists contains articles on many of the points above.

Software and code

Policy information about availability of computer code

Data collection	No software was used for data collection as data is publicly available for download from the Web of Life ecological networks database.
Data analysis	Simulations and all data analysis was conducted in R (ver 4.0.2). All code necessary to reproduce the results are publicly accessible from Zenodo: http://doi.org/10.5281/zenodo.4749405

For manuscripts utilizing custom algorithms or software that are central to the research but not yet described in published literature, software must be made available to editors and reviewers. We strongly encourage code deposition in a community repository (e.g. GitHub). See the Nature Research guidelines for submitting code & software for further information.

Data

Policy information about availability of data

All manuscripts must include a data availability statement. This statement should provide the following information, where applicable:

- Accession codes, unique identifiers, or web links for publicly available datasets
- A list of figures that have associated raw data
- A description of any restrictions on data availability

All data are available in the main text or supplementary material. Empirical network data is publicly available from the Web of Life ecological networks database (http://www.web-oflife.es). Fig. 4 has associated raw data from the Web of Life database, with data pre-processing described in full in the Methods section.

Field-specific reporting

Please select the one below that is the best fit for your research. If you are not sure, read the appropriate sections before making your selection.

☐ Life sciences ☐ Behavioural & social sciences ☒ Ecological, evolutionary & environmental sciences

For a reference copy of the document with all sections, see nature.com/documents/nr-reporting-summary-flat.pdf

Ecological, evolutionary & environmental sciences study design

All studies must disclose on these points even when the disclosure is negative.

Study description	This study uses simulated bipartite networks and theory to generate a parameter for predicting the vulnerability of networks to species or functional trait loss. Theoretical results are tested on empirical bipartite network data from the Web of Life database (http://www.web-oflife.es).
Research sample	Generated data are simulations produced in R. Additional empirical data comes from the Web of Life database (source: http://www.web-oflife.es). This empirical data is comprised of Anemone-Fish (n = 17), Host-Parasite (n = 51), Plant-Ant (n = 4), Plant-Pollinator (n = 148) and Seed-Disperser (n = 34) bipartite networks. Empirical data was chosen because these are bipartite networks representing real-world empirical systems.
Sampling strategy	Simulations were generated across a range of sample sizes to determine the generality of findings. Our choice of sample sizes was representative of a wide range of systems from 'simple' ecosystem services unpinned by few functions and species, to complex systems representing more complex ecosystem services provided by a large network of species and functions (traits). Empirical network data was excluded if sample size was insufficient (i.e. three networks had only one 'trait' on one half of the bipartite network).
Data collection	Data simulations were conducted by Jean-Francois Arnoldi and Andrew Jackson, and empirical network data was downloaded from the Web of Life database by Andrew jackson. Data were saved in .csv format.
Timing and spatial scale	Data is simulated to represent a bipartite species-traits network at a single point in time (that is, temporal scale is not explicitly considered or a relevant notion here). Simulations were conducted on 29 January 2020. Spatial scale is not explicitly considered, but the bipartite networks are assumed to be fully-resolved, making the spatial scale of relevance the community network scale.
Data exclusions	Exclusion criteria were pre-established to select only those bipartite networks (from the Web of Life database) which were suitable to test our hypothesis and to measure our parameters of interest: "We removed three networks that comprised only a single trait (that is, where all species connected to the same single interaction partner), and four that showed no variation in the number of species per trait (of which the latter were all Anemone-Fish webs), leaving 251 networks for analysis."
Reproducibility	Simulations are entirely reproducible based on the formulae provided in the manuscript and using the publicly available code. Simulations covered a range of sample sizes of all parameters to prevent basing results on spurious findings.
Randomization	Simulated species loss was conducted randomly, and simulated boolean networks were generated randomly based on pre-determined parameters.
Blinding	Simulations were exploratory in nature in order to identify parameters of relevance for understanding patterns of species loss and network vulnerability. As such, blinding was neither possible nor relevant during the simulated part of the study. Empirical networks were analysed blind, using code to calculate the appropriate network parameters irrespective of the identity of the network. Network dispersion (Fig. 3a) and network type (Fig. 3d) were then analysed post-hoc, meaning blinding was not possible or relevant.

Did the study involve field work? ☐ Yes ☒ No

Reporting for specific materials, systems and methods

We require information from authors about some types of materials, experimental systems and methods used in many studies. Here, indicate whether each material, system or method listed is relevant to your study. If you are not sure if a list item applies to your research, read the appropriate section before selecting a response.

Materials & experimental systems

n/a	Involved in the study
☒	☐ Antibodies
☒	☐ Eukaryotic cell lines
☒	☐ Palaeontology and archaeology
☒	☐ Animals and other organisms
☒	☐ Human research participants
☒	☐ Clinical data
☒	☐ Dual use research of concern

Methods

n/a	Involved in the study
☒	☐ ChIP-seq
☒	☐ Flow cytometry
☒	☐ MRI-based neuroimaging
